# Functional characterization of a 3-O-sulfate tolerant heparin lyase from *Bacteroides eggerthii*

**DOI:** 10.1093/glycob/cwag097

**Published:** 2026-10-13

**Authors:** Taran K Kandola, Courtney J Mycroft-West, Marcelo A Lima, Abigail Turner, Cristina Gardini, Elena Urso, Thomas A Torode, Gavin J Miller, Antonella Bisio, Edwin A Yates, Marco Guerrini, Mark A Skidmore, Liang Wu

**Affiliations:** Centre for Marine and Coastal Studies (CEMACS), Universiti Sains Malaysia, 11800 USM, Penang, Malaysia; Centre for Glycoscience Research and Training, Keele University, Newcastle-under-Lyme, Staffordshire, ST5 5BG, United Kingdom; The Rosalind Franklin Institute, Harwell Science and Innovation Campus, OX11 0QX, United Kingdom; Department of Biochemistry, University of Oxford, South Parks Road, Oxford, OX1 3QU, United Kingdom; Centre for Glycoscience Research and Training, Keele University, Newcastle-under-Lyme, Staffordshire, ST5 5BG, United Kingdom; The Rosalind Franklin Institute, Harwell Science and Innovation Campus, OX11 0QX, United Kingdom; Department of Biochemistry, University of Oxford, South Parks Road, Oxford, OX1 3QU, United Kingdom; Centro Alta Tecnologia Istituto di Ricerche Chimiche e Biochimiche ‘G. Ronzoni’ S.r.l., Via G. Colombo 81, 20133, Milan, Italy; Istituto di Ricerche Chimiche e Biochimiche ‘G. Ronzoni’, Via G. Colombo 81, 20133, Milan, Italy; Centre for Glycoscience Research and Training, Keele University, Newcastle-under-Lyme, Staffordshire, ST5 5BG, United Kingdom; Centre for Glycoscience Research and Training, Keele University, Newcastle-under-Lyme, Staffordshire, ST5 5BG, United Kingdom; Manchester Institute of Biotechnology, University of Manchester, 131 Princess Street, Manchester, M1 7DN, United Kingdom; Istituto di Ricerche Chimiche e Biochimiche ‘G. Ronzoni’, Via G. Colombo 81, 20133, Milan, Italy; Centre for Glycoscience Research and Training, Keele University, Newcastle-under-Lyme, Staffordshire, ST5 5BG, United Kingdom; Istituto di Ricerche Chimiche e Biochimiche ‘G. Ronzoni’, Via G. Colombo 81, 20133, Milan, Italy; Department of Biochemistry, Cell and Systems Biology, Institute of Structural, Molecular and Integrative Biology, University of Liverpool, Crown Street, Liverpool, L69 7ZB, United Kingdom; Istituto di Ricerche Chimiche e Biochimiche ‘G. Ronzoni’, Via G. Colombo 81, 20133, Milan, Italy; Centre for Marine and Coastal Studies (CEMACS), Universiti Sains Malaysia, 11800 USM, Penang, Malaysia; Centre for Glycoscience Research and Training, Keele University, Newcastle-under-Lyme, Staffordshire, ST5 5BG, United Kingdom; Department of Biochemistry, Cell and Systems Biology, Institute of Structural, Molecular and Integrative Biology, University of Liverpool, Crown Street, Liverpool, L69 7ZB, United Kingdom; The Rosalind Franklin Institute, Harwell Science and Innovation Campus, OX11 0QX, United Kingdom; Department of Pharmacology, University of Oxford, Mansfield Road, Oxford, OX1 3QT, United Kingdom

**Keywords:** anticoagulation, enzyme, heparin, lyase

## Abstract

Heparin is a sulfated glycosaminoglycan, and the most used pharmaceutical anticoagulant worldwide. Heparin is isolated primarily from porcine sources, and possesses a complex variable structure, wherein anticoagulant activity is principally derived from the pentasaccharide GlcNS6S-GlcA-GlcNS3S6S-IdoA2S-GlcNS6S (AGA^*^IA) motif, containing an infrequent 3-O-sulfated glucosamine that enhances antithrombin stabilization. *Pedobacter heparinus* heparin lyases are currently widely employed for characterization of heparin and its derivatives, but have limited activity near regions of 3-O-sulfation, hindering analyses of anticoagulation relevant sequences. Here, using a panel of biophysical and structural techniques, we show that *Bacteroides eggerthii* lyase I liberates ΔUA-GlcNS3S6S disaccharides from the defined pentasaccharide, fondaparinux. This activity provides improved access to 3-O-sulfated heparin standards, with potential to aid heparin quality control.

## Introduction

Heparin, a highly sulfated glycosaminoglycan (GAG) polysaccharide, is a WHO Essential Medicine that provides anticoagulant activity for the treatment and prophylaxis of thrombotic disorders (Hemker 2016; Hogwood et al. 2023). Currently, the structural complexity of heparin polysaccharides precludes chemical synthesis, thus it continues to be derived from natural sources, with corresponding batch-to-batch variability. Pharmaceutical heparin today is primarily of porcine origin (porcine mucosal heparin; PMH), but concerns over global supply, and religious/societal preferences, are driving exploration of alternative sources e.g. bovine/ovine (Tovar et al. 2016; Al-Hakim 2021). Shifts to alternative heparins introduce species-related complexities, underscoring the need for standardization to ensure safety and efficacy of this essential pharmaceutical agent.

Compositionally, heparin is related to another GAG, heparan sulfate (HS) (Meneghetti et al. 2015), which is produced by virtually all metazoan cell types, and mediates extracellular functions including cell signaling, differentiation, migration, adhesion and endocytosis (Sarrazin et al. 2011). Structurally, heparin and HS are linear chains of 1 → 4 linked disaccharide units, comprising N-acetyl- or N-sulfo-glucosamines (α-D-GlcNAc or α-D-GlcNS), alternating with glucuronic or iduronic acids (β-D-GlcA or α-L-IdoA). These monosaccharide units may be further modified by *O*-sulfation at O2 of IdoA, O6 of GlcNS, and (less frequently) O3 of GlcNS (Fig. 1a) (Rabenstein 2002).

**Figure 1:**
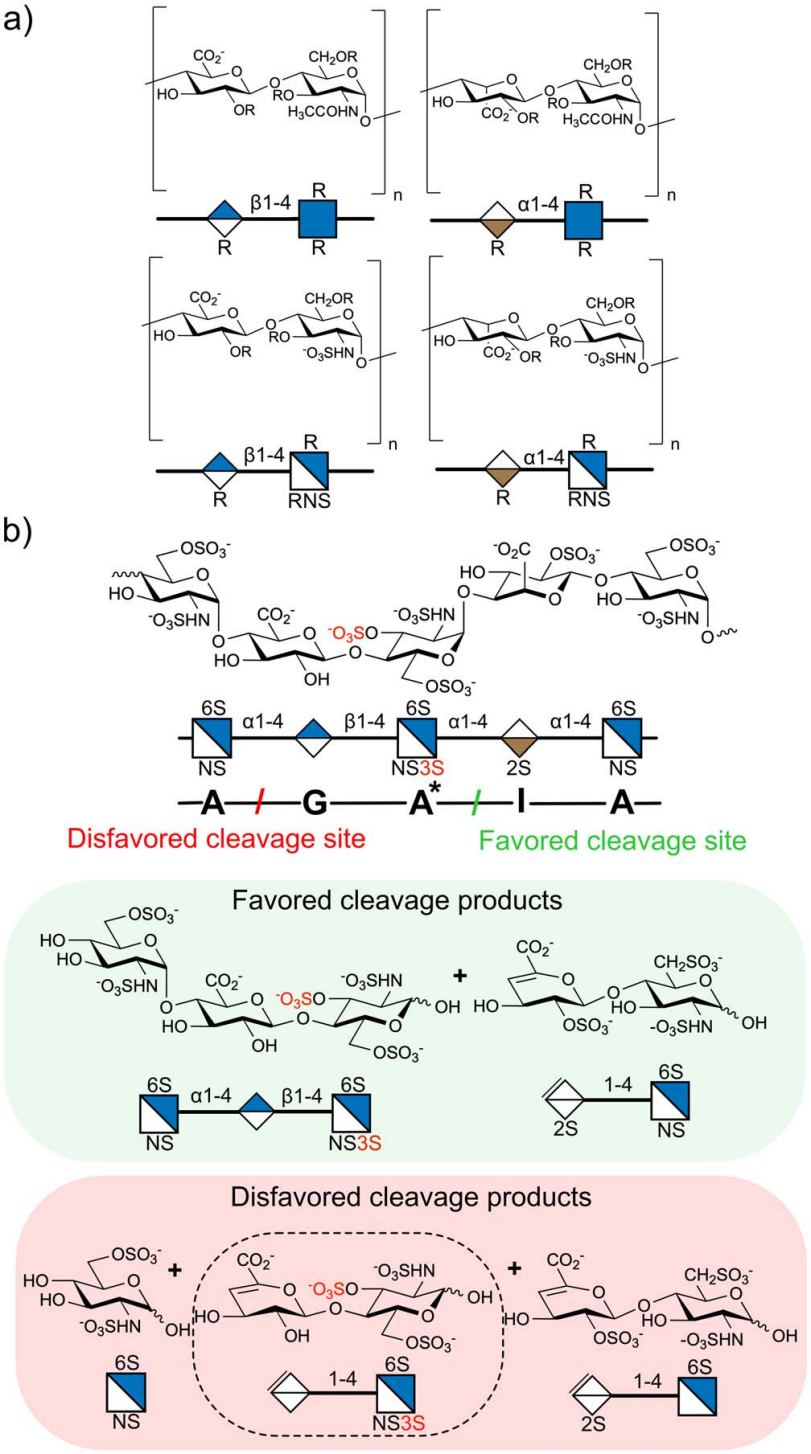
a) Major disaccharide repeating units of heparin and HS. R = H or SO_3_^-^. b) Pentasaccharide AGA^*^IA - principally responsible for heparin anticoagulant activity, is cleaved by *Ph*HepI at AGA^*↓^IA. Cleavage at A^↓^GA^*^IA is disfavored.

Heparin exerts its anticoagulant properties through the inhibition of blood coagulation proteases (primarily factors IIa [thrombin] and Xa) by binding to, and enhancing, the activity of antithrombin III (AT) (Bjork and Lindahl 1982). Canonically, the heparin pentasaccharide GlcNS6S-GlcA-GlcNS3S6S-IdoA2S-GlcNS6S (AGA^*^IA, where ^*^ denotes 3-*O*-sulfation) binds to AT with high (~3 nM) affinity, inducing structural changes that enhance its protease inhibition (Fig. 1b) (Atha et al. 1985). 3-*O*-sulfation of AGA^*^IA improves AT activation ~1000-fold compared to AGAIA, and is thus a key modification for heparin anticoagulant activity (Atha et al. 1985). Due to its complexity, accurate quantification of 3-O-sulfation in heparin preparations is a non-trivial endeavor, but remains essential for pharmaceutical characterization, and will become increasingly important as new heparin sources are approved for market.

Primary methods for heparin/HS characterization largely rely upon polysaccharide depolymerization by β-eliminative lyases, followed by chromatographic analysis of the resultant disaccharide constituents, with comparison to known standards. *Pedobacter heparinus* heparin lyases I-III (*Ph*HepI-III) are currently the most widely used enzymes for heparin/HS depolymerization, but display very limited cleavage at uronic acids to the non-reducing end of 3-*O*-sulfated glucosamines (e.g. A^↓^GA^*^IA) (Fig. 1b) (Yamada et al. 1993; Shriver et al. 2000). Disaccharides containing 3-*O-*sulfates cannot therefore be generated reliably using *Ph*Heps, hindering access to analytical standards containing this modification. Attempts to circumvent this fundamental limitation of *Ph*Heps in heparin characterization have included the use of isotopically labelled digest standards (Wang et al. 2022; Wang et al. 2023), tetrasaccharide heparin standards (Mochizuki et al. 2025), 3O-sulfatase co-digestion (Poyer et al. 2026), or digesting oligosaccharides with extremely large amounts of enzyme (Karlsson et al. 2021).

Many *Bacteroides* strains have evolved to process complex carbohydrates within animal digestive tracts as sources of carbon and energy, including GAGs from dietary and host-cell sources. Heparinase I from *Bacteroides eggerthii* (*Be*HepI) was previously shown to exhibit broad specificity against heparin uronic acids (Liu et al. 2020). Herein, *Be*HepI heparin depolymerization was evaluated against *Ph*HepI using biophysical and structural approaches, revealing improved suitability of the *Bacteroides* enzyme for 3-O-sulfated disaccharide preparation, with potential for use as an additional tool in heparin structural analysis.

## Results and discussion

Recombinant *Be*HepI was expressed and purified (Fig. S1), and its depolymerization of PMH assessed by monitoring Δλ_abs_ = 232 nm, corresponding to the unsaturated alkene generated by lyase cleavage of heparin/HS uronic acids (Fig. 2a). Apparent Michaelis–Menten kinetics indicated efficient PMH digestion by *Be*HepI, with k_cat_ = 1.3 s^−1^ and K_M_ = 37.7 μg.mL^−1^ (~65 μM, assuming an “average” disaccharide substrate of 578 Da mass (Gatti et al. 1979)). Size exclusion chromatography of PMH treated exhaustively with *Be*HepI or *Ph*HepI showed that products of *Be*HepI digestion were predominantly <3.6 kDa in size, whilst *Ph*HepI yielded higher molecular weight material (4.2–6.6 kDa) (Fig. 2b), indicating improved depolymerization by *Be*HepI.

**Figure 2:**
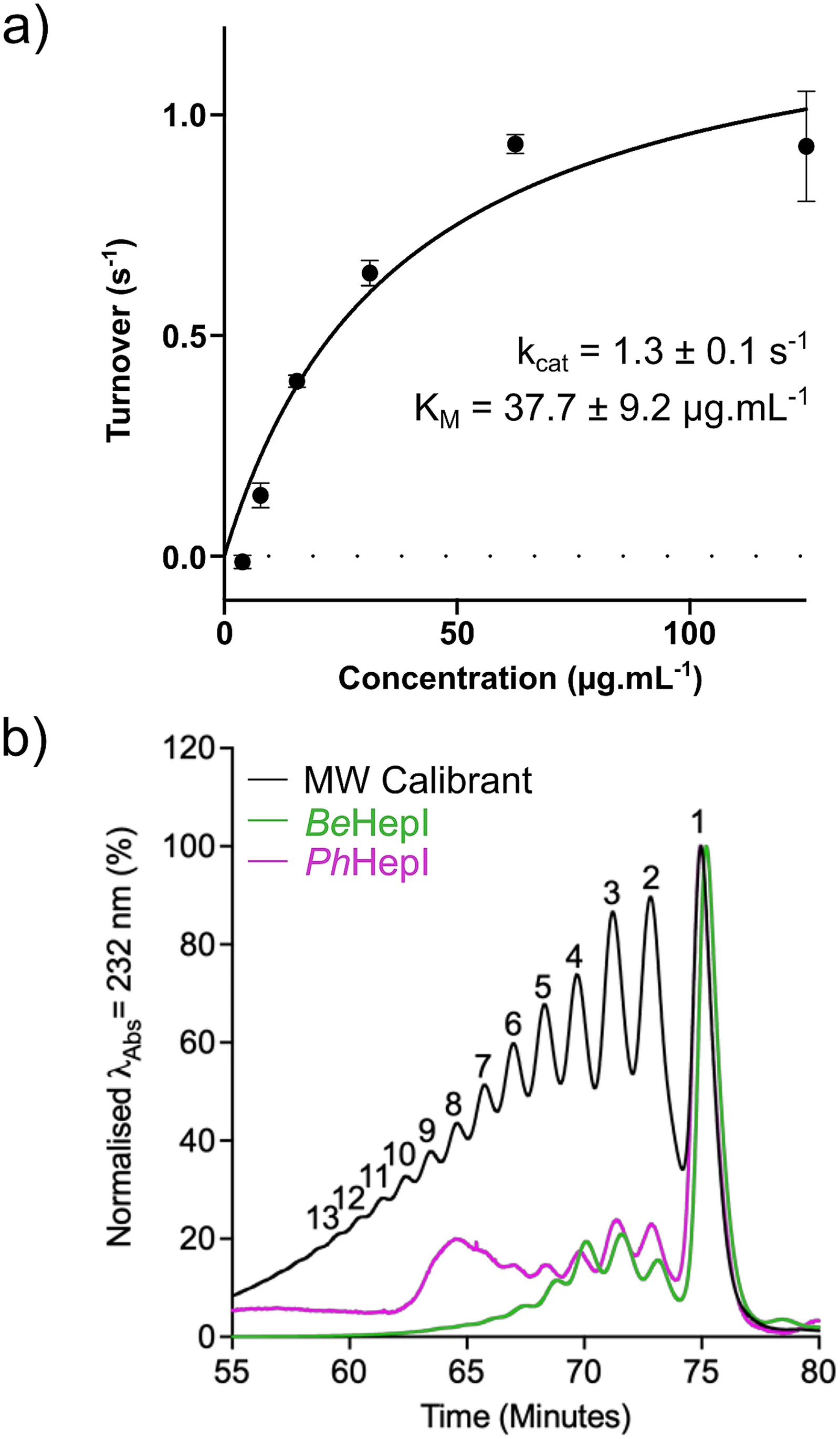
a) Michaelis–Menten kinetics of PMH digestion by *Be*HepI, monitoring λ_Abs_ = 232 nm. Datapoints are mean ± s.e.m. from 3 technical replicates. b) Size exclusion chromatogram of PMH, digested exhaustively with *Be*HepI or *Ph*HepI. Molecular weight calibrants 1–13 correspond to 0.6–8.4 kDa (0.6 kDa increments).

To dissect the cleavage specificities of *Be*HepI vs *Ph*HepI, the pentasaccharide fondaparinux sodium (Arixtra®), a fully synthetic heparin mimetic comprising reducing end *O*-methylated AGA^*^IA, was employed as a defined substrate. LC–MS analysis of fondaparinux treated with *Be*HepI showed ions at m/z 337.99 (−1) (assignment A1,2,0), m/z 575.96 (−1) (ΔU2,3,0), m/z 705.12 (−1) (ΔU2,3,0 dibutylamine adduct), and m/z 589.98 (−1) (ΔU2,3,0-OMe), consistent with cleavage at both A^↓^GA^*↓^IA-OMe to liberate A, GA^*^ and IA-OMe respectively (Figs. 3a and b). In contrast, only one disaccharide corresponding to IA-OMe (m/z 589.98 (−1) ΔU2,3,0-OMe) was detected following fondaparinux treatment with *Ph*HepI, indicating single cleavage at AGA^*↓^IA-OMe (Figs. 3c and d; peak 4). The m/z 456.97 (−2) ion (A3,5,0), corresponding to trisaccharide AGA^*^, was also more abundant following *Ph*HepI digestion compared to *Be*HepI, and no monosaccharide m/z 337.99 (−1) (A1,2,0) could be observed (Figs. 3a and c; peak 6; Tables S1 and S2).

**Figure 3:**
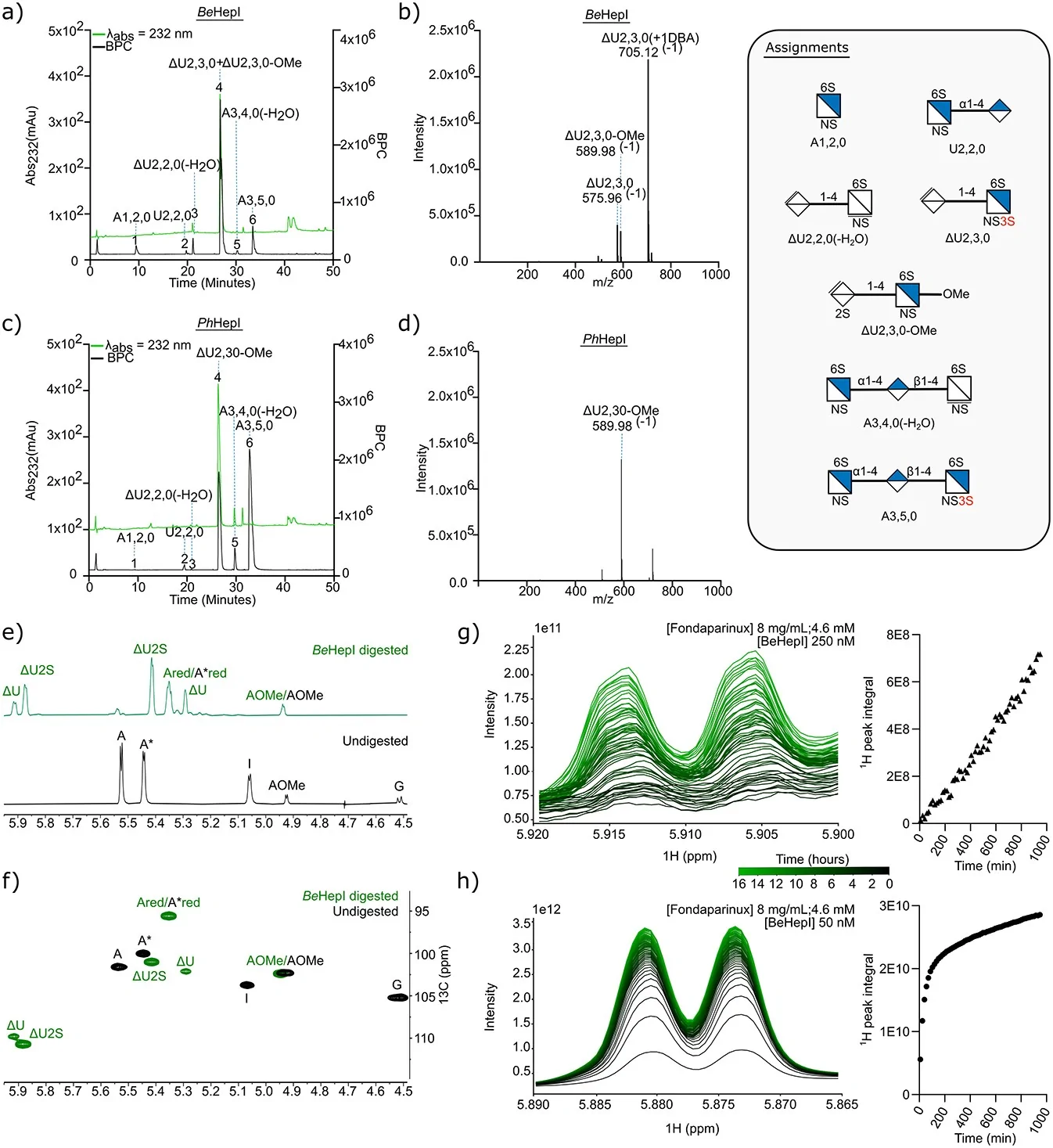
a) LC–MS base-peak chromatogram (BPC) trace and λ_Abs_ = 232 nm of fondaparinux digestion fragments produced by *Be*HepI. b) Mass spectrum corresponding to peak 4 from a. c) LC–MS BPC and λ_Abs_ = 232 nm of major fondaparinux digestion fragments produced by *Ph*HepI. d) Mass spectrum corresponding to peak 4 from c. e) ^1^H and f) ^1^H-^13^C HSQC NMR spectra of fondaparinux before and following exhaustive digestion with *Be*HepI. g) ^1^H NMR timecourse of fondaparinux ΔUA ^1^H peak formation from A↓GA^*^IA. h) ^1^H NMR timecourse of ΔUA2S ^1^H peak formation from AGA^*↓^IA.

^1^H NMR spectroscopy was next employed to examine the enzymatic digestion of fondaparinux by *Be*HepI, comparing spectra of the pentasaccharide before and after digestion. Two alkene proton signals were observed following fondaparinux enzyme treatment (5.89–5.92 ppm), corresponding to unsaturated products formed by both A^↓^GA^*↓^IA cleavage (Fig. 3e) (Limtiaco et al. 2011). 2D ^1^H-^13^C NMR also showed expected chemical shifts for the anomeric peaks of IdoA2S (5.07/103 → 5.42/101 ppm) and GlcA (4.52/106 → 5.29/102 ppm) (Fig. 3f); structures of digestion products were fully assigned using COSY and TOCSY 2D NMR (Figs. S2-S4). Quantitation of ΔUA ^1^H peaks over time revealed that the AGA^*↓^IA-OMe site was cleaved substantially more rapidly than A^↓^GA^*^IA-OMe, suggesting that whilst *Be*HepI does efficiently digest uronic acids to the non-reducing end of 3-*O*-sulfated glucosamines, other sites may still be preferred (Figs. 3g and h, S5).

Having mapped *Be*HepI digestion of model pentasaccharide fondaparinux, we sought to examine its activity against more complex substrates. First, comparison of *Be*HepI and *Ph*HepI against a longer defined decasaccharide containing 4 uronic acid cleavage sites, including one adjacent to 3-O-sulfated glucosamine, showed that *Be*HepI cleaved all substrate bonds, contrasting to largely single cleavage by *Ph*HepI, reinforcing observations from fondaparinux (Fig. S6, Tables S3,4). Next, we tested digestion of a bulk PMH preparation previously confirmed to contain 3-O-sulfated residues (Santini et al. 1997). Using NMR, we observed that *Be*HepI digestion produced ^1^H-^13^C peaks at 5.4/94.3 ppm in HSQC spectra, corresponding to newly liberated A1 reducing ends (Ared; Fig. S7a). TOCSY showed coupling of these Ared protons to both 3.44 ppm (A2^*^) and 4.47 ppm (A3^*^) peaks, indicating linkage to 3-O-sulfated glucosamines (Figs. S7b and c). Multiple overlapping ΔUA peaks were observed across 5.9–6.4 ppm, consistent with broad *Be*HepI activity against uronic acids from many contexts (Fig. S7a). Intriguingly, LC–MS analyses also showed loss of several 3-O-sulfated tetrasaccharides/hexasaccharides following PMH digestion by *Be*HepI vs *Ph*HepI, consistent with improved cleavage activity against these fragments (Fig. S8, Tables S5,6). Taken together, our data support the ability of *Be*HepI to digest uronic acids near 3-O-sulfated glucosamines, even within complex PMH samples.

Finally, to understand the structural basis of *Be*HepI activity towards heparin/HS, X-ray crystal structures were obtained for the enzyme in both apo form (PDB 9GRW, Table S7) and following treatment with fondaparinux (PDB 9GV9). Isomorphous datasets for apo and fondaparinux treated *Be*HepI were processed to 1.7 Å and 1.9 Å respectively (Fig. S9). Each structure contained two protein molecules in the asymmetric unit, with a small (~819 Å^2^) contact interface consistent with crystallographic packing.

The 3-dimensional structure of apo *Be*HepI resembles that of a previously reported *Bacteroides thetaiotaomicron* homolog (*Bt*HepI; PDB 3IKW; RMSD 1.9 Å over 366 amino acids; 84% identity) (Han et al. 2009), with a central β-jelly roll fold (residues 28–111, 139–175, 242–391), extended Ω-loop (112–138), and “thumb” domain (176–241). The inner antiparallel β-sheet of the β-jelly roll and the thumb domain form a positively charged substrate cleft, and are connected through the sidechains of Glu240 and Asp364, the main chain carbonyls of Trp266 and Asn363, and an octahedrally coordinated Ca^2+^ (Figs. 4a, S10).

**Figure 4:**
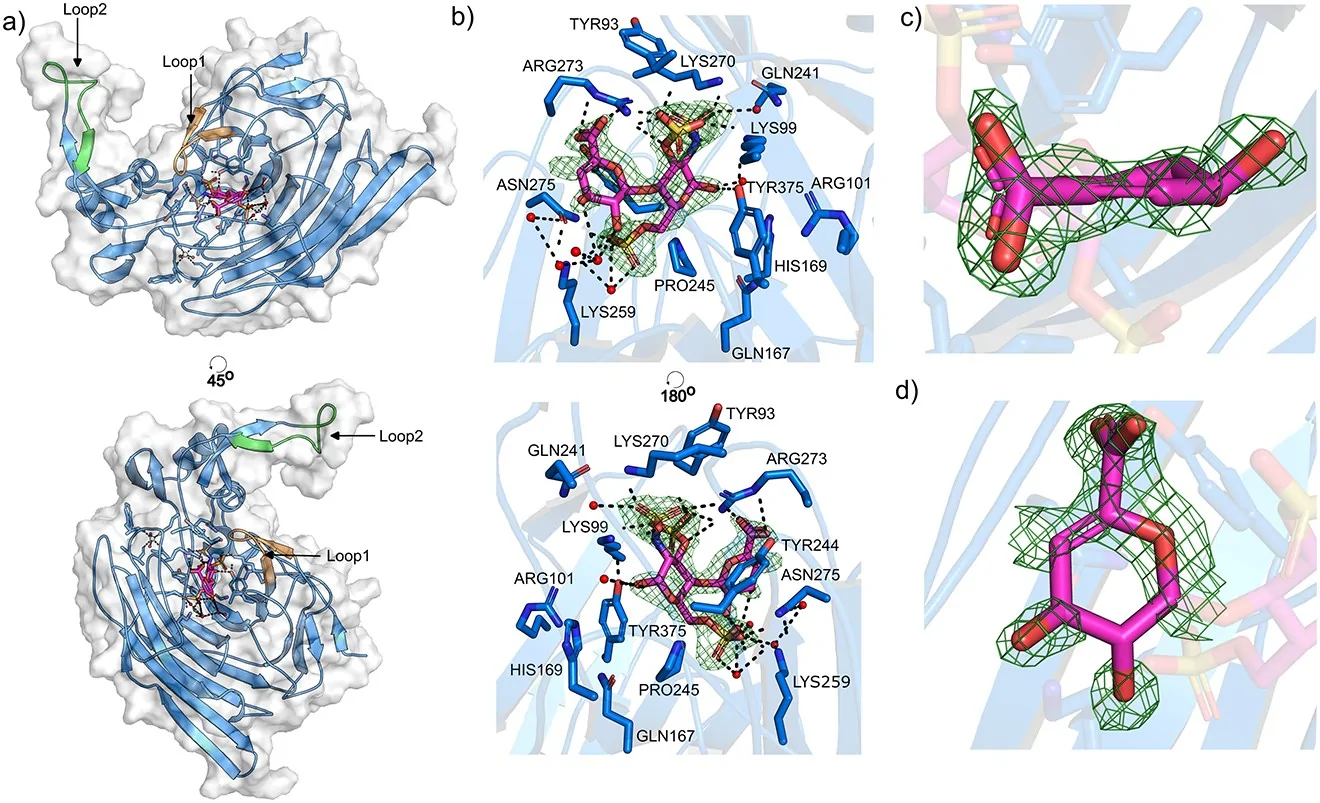
a) Structure of *Be*HepI bound to GA^*^. b) Active site views in complex with GA^*^ disaccharide product in the −1 and − 2 subsites. c) Close-up views of the unsaturated C4-C5 bond resulting from β-elimination cleavage. d) Strong electron density around the extended C4–C5–(C6)–O5 π-system. Electron density for ligand is REFMAC σ_A_-weighted mFo-DFc, contoured to 2σ (0.25 e^−^/Å^3^).

The structure of *Be*HepI reacted with fondaparinux showed clear difference density within the enzyme catalytic centre, which could be well-modelled by an unsaturated GA^*^ disaccharide across both −1, −2 subsites, indicating a product complex arising from fondaparinux cleavage (Phenix map-to-model correlation 0.94; Fig. 4b). GA^*^ engaged *Be*HepI with its reducing-end hydroxyl coordinated to Tyr375, *N*-sulfate coordinated to Lys270, Gly223 and Arg253, 3-*O*-sulfate coordinated to Tyr93 and Lys99, 6-*O*-sulfate coordinated to Asn275 and Lys259, and ΔUA carboxylate coordinated to Arg273 and Tyr244. Notably, electron density for GA^*^ within the *Be*HepI active site was highly planar across the C3–C4–C5 region, in line with β-eliminative formation of a π-bond (Fig. 4c). Electron density around C4–C5–(C6)–O5 was also stronger compared to other areas of the ring, consistent with an extended π-system encompassing the alkene, endocyclic oxygen, and C6 carboxylate (Figs. 4d, S11). These structural data support LC–MS and NMR assessments, confirming the ability of *Be*HepI to cleave A^↓^GA^*↓^IA fondaparinux bonds.

Structural comparison of *Be*HepI and *Ph*HepI suggested a possible mechanistic basis for their differing cleavage efficiencies. No experimental structure currently exists for *Ph*HepI. However, AlphaFold prediction (Abramson et al. 2024) indicates that *Ph*HepI is likely to adopt a similar 3-dimensional fold to *Be*HepI, with principal differences arising from two loops around the substrate binding cleft; Leu90-Lys99 (loop 1; *Be*HepI numbering) and Ala211-Lys230 (loop 2) (Fig. 4a). Lys95 in *Be*Hep loop 1 corresponds to Ala41 in *Ph*HepI, which contributes no positive charge across the active site. Loop 2 of *Be*HepI also contains an eight amino acid extension compared to *Ph*HepI, forming an elongated cationic lid that covers its active site. These differences may rationalize (in part) the improved activity of *Be*HepI against more negatively charged heparin/HS oligosaccharides carrying 3-*O*-sulfation (Fig. S12).

In summary, *Be*HepI displays distinct heparin/HS substrate specificities compared to currently used *Ph*Heps, including efficient cleavage of uronic acid residues towards the non-reducing end of 3-O-sulfated glucosamines (positions that are poorly processed by *Ph*Heps). Such activity likely renders *Be*HepI better suited to the preparation of standards containing 3-O-sulfation, which will aid analyses of biologically important heparin/HS sequences. Whilst we have not unequivocally ascertained here whether *Be*HepI by itself can liberate 3-O-sulfated disaccharides from PMH, the broad activity of this enzyme is likely to benefit multistep depolymerization strategies. Accordingly, *Be*HepI offers routes towards heparin/HS analysis, beyond what is currently tractable using *Ph*Heps alone.

## Methods

### Expression and purification of *Be*HepI

Heparinase I from *B. eggerthii* (WP_004294077.1), lacking the N-terminal leader sequence (21–394), was inserted into pET-22b(+) (Merck, UK) and transformed into OverExpress™ C41 (DE3) pLysS cells (Imaxio SA). Transformed cells were grown to OD_600_ 0.6–0.8, and induced with 1 mM IPTG. For protein purification, *Be*HepI cell pellets were resuspended in 25 mM Tris–HCl, 500 mM NaCl, 5 mM CaCl_2_, 20 mM imidazole pH 7.4, supplemented with cOmplete™ protease inhibitor (Merck) and DNase I (Merck). Cells were lysed using a cell disruptor (Constant Systems), and clarified by centrifugation (16,000 g; 30 min; 4 °C). Initial protein capture was carried out by using a HisTrap FF column (Cytiva), employing a linear gradient of 25 mM Tris, 500 mM NaCl, 5 mM CaCl2, pH 7.4, 20➔200 mM imidazole. Further polishing was conducted by size-exclusion (Cytiva S75 16/600; 25 mM Tris, 100 mM NaCl, 5 mM CaCl_2_ pH 7.4 buffer) and ion-exchange (Cytiva Resource S; 20 mM MES, 5 mM CaCl_2_, pH 6.0, 0➔1500 mM NaCl). Purified *Be*HepI was flash-frozen in liquid nitrogen and stored at −80 °C for further use.

### *Be*HepI kinetic assay

A 6-point 2-fold serial dilution of PMH (Celsus, USA) from 125 μg.mL^−1^ was incubated with 5 nM *Be*HepI in 100 mM sodium acetate and 0.1 mM calcium acetate, pH 7.0, at 30 °C. 200 μL reactions were performed in triplicate in 96-well UV-STAR® microplates (Greiner) with λ_abs_ = 232 nm monitored over 20 minutes using a Clariostar plate reader (BMG Labtech; v5.70R2). Controls omitting *Be*Hep1 were subtracted for analysis. The extinction coefficient of ΔUA at 232 nm (5550 M^−1^ cm^−1^), was used to calculate product concentration. Initial reaction velocities at each substrate concentration were fitted to the Michaels Menten equation in Prism (Graphpad; v11.1) to derive *K_m_*, and *k_cat_*.

### Exhaustive enzymatic digestion

Fondaparinux (Sigma-Aldrich, SML1240), decasaccharide, or PMH was treated with *Be*HepI or *Ph*HepI in 100 mM sodium acetate and 0.1 mM calcium acetate, pH 7.0 for 48 h at 37 °C under constant agitation. Equal quantities of enzyme were added at 0, 1, 2 and 24 h, totalling 160 mIU. After 48 h the reaction was heat inactivated (95 °C for 2 min), filtered (0.22 μm cellulose acetate; LLG labware) and analysed by chromatography, LC–MS, or NMR, unless stated otherwise.

### Analytical size exclusion chromatography

Oligosaccharide profiling of PMH digestion products was performed using gel permeation chromatography (TSKG3000SW, 7.8 mm(ID)×30 cm, 5.0 μm and TSKG2000SW, 7.8 mm(ID)×30 cm, 5.0 μm; columns connected in series; Tosoh Bioscience GmbH) with a mobile phase of 0.30 M sodium sulfate, on a HPLC system measuring λ_abs_ = 232 nm (Agilent). Isocratic elution was conducted at 300 μL.min^−1^ and peaks were compared to Heparin Low-Molecular-Mass for Calibration CRS standards (EDQM).

### Liquid chromatography-mass spectrometry

LC–MS was performed on digested samples using a reverse phase HPLC system (Platin Blue, Knauer) coupled to a Bruker ESI- Q-TOF Impact II Daltonics system. Ion-pair reverse phase separation was achieved by C-18 Kinetex column (100x2.1 mm, 2.6 μm, 100 Å; Phenomonex), applying a multi-step gradient elution with Phase A (10 mM dibutylamine, 10 mM acetic acid in water) and Phase B (10 mM dibutylamine, 10 mM acetic acid in methanol) with a flow rate of 0.15 ml.min^−1^ at 35 °C. 8 μg sample was injected for each heparinase I digest onto the LC–MS setup, with MS parameters for analysis: ESI in negative ion mode; capillary voltage +3500 V; nebulizer pressure 1.8 bar; dry gas flow 7.0 L.min^-1;^ dry gas temperature 200 °C, m/z scan range 140–2500.

### Nuclear magnetic resonance

1-D (^1^H), 2-D (^1^H-^13^C) HSQC, 2-D COSY and 2-D TOCSY spectra of exhaustive digests were recorded using a Bruker Avance NEO 600 MHz spectrometer fitted with a 5 mm QCIF cryoprobe, at 298 K. NMR spectra of partial fondaparinux digests were recorded on a Bruker Avance 400 MHz spectrometer equipped with a BBFO probe. 1-D and 2-D NMR spectra were collected using standard Bruker pulse sequences.

Time-course digestion of 2 mg fondaparinux sodium by *Be*HepI (50 or 250 nM) was performed at 303 K in a reaction buffer comprising 100 mM sodium acetate and 0.1 mM calcium acetate, pH 7.0 and 10% D_2_O with a total volume of 250 μL. Reactions were carried out in a Bruker Avance NEO 600 MHz spectrometer fitted with a 5 mm QCIF cryoprobe (Bruker, UK), with shimming, proton pulse width calibration and solvent suppression calibration performed using a sample omitting *Be*HepI. Following addition and mixing of *Be*HepI (dead time 2 min), polysaccharide digestion was monitored by recording back-to-back perfect echo watergate 1-D (^1^H) spectra (Adams et al. 2013) with an experiment time of 33 s, for 16 h (experimental parameters: ns = 8, ds = 4, d1 = 1 s, aq = 1.7 s). Spectra were processed with an exponential window function, Fourier transformed and phased in NMRPipe (Delaglio et al. 1995), before being imported into python 3.11 using nmrglue (Helmus and Jaroniec 2013). Every 30 s, ^1^H spectra were summed and calibrated internally to acetate (1.82 ppm). Spectral windows corresponding to unsaturated disaccharide products (5.8–5.6 ppm) (Limtiaco et al. 2011) were plotted in Jupyter Notebook using matplotlib. Peak areas were quantified by integration over 5.900–5.920 or 5.89–5.865 ppm regions, using the trapezoidal rule following linear baseline correction.

### Crystallography

Well-diffracting crystals of *Be*HepI were grown and cryo-harvested from 2 M (NH_4_)_2_SO_4_, 0.1 M CH_3_CO_2_Na, pH 5.0. Ligand complexes with fondaparinux were obtained by addition of ligand powder directly into drops containing crystals, followed by incubation for 5 minutes before harvesting. Diffraction data was collected at Diamond Light Source (UK). Liganded and apo datasets were processed with STARANISO (Tickle et al. 2016) and xia2 (Gildea et al. 2022) pipelines respectively. Structures were solved by molecular replacement with MOLREP (Vagin and Teplyakov 2010), using PDB 3IKW as a search model, before iterative rounds of manual model building with COOT (Casanal et al. 2020), alongside maximum-likelihood refinement with REFMAC5 (Murshudov et al. 2011). Ligand coordinates and dictionaries were defined using JLigand (Lebedev et al. 2012). Structure figures were generated using Pymol.

## Supplementary Material

Rev1_supp_behepI_cwag097(1)

## Data Availability

Crystal structure coordinates and electron density maps have been deposited under PDB accession codes 9GRW (apo *Be*HepI) and 9GV9 (*Be*HepI fondaparinux).

## Notes

This accepted manuscript is for the SPECIAL ISSUE: SfG 2025 Meeting"

## References

[ref1] Abramson J, Adler J, Dunger J, Evans R, Green T, Pritzel A, Ronneberger O, Willmore L, Ballard AJ, Bambrick J, et al. Accurate structure prediction of biomolecular interactions with AlphaFold 3. Nature. 2024:630(8016):493–500. 10.1038/s41586-024-07487-w. PMC11168924 38718835

[ref2] Adams RW, Holroyd CM, Aguilar JA, Nilsson M, Morris GA. "perfecting" WATERGATE: clean proton NMR spectra from aqueous solution. Chem Commun (Camb). 2013:49(4):358–360. 10.1039/C2CC37579F. 23192194

[ref3] Al-Hakim A . General considerations for diversifying heparin drug products by improving the current heparin manufacturing process and reintroducing bovine sourced heparin to the US market. Clin Appl Thromb Hemost. 2021:27:10760296211052293. 10.1177/10760296211052293. PMC8678904 34894779

[ref4] Atha DH, Lormeau JC, Petitou M, Rosenberg RD, Choay J. Contribution of monosaccharide residues in heparin binding to antithrombin III. Biochemistry. 1985:24(23):6723–6729. 10.1021/bi00344a063. 4084555

[ref5] Bjork I, Lindahl U. Mechanism of the anticoagulant action of heparin. Mol Cell Biochem. 1982:48(3):161–182. 10.1007/BF00421226. 6757715

[ref6] Casanal A, Lohkamp B, Emsley P. Current developments in Coot for macromolecular model building of electron Cryo-microscopy and crystallographic data. Protein Sci. 2020:29(4):1069–1078. 10.1002/pro.3791. PMC7096722 31730249

[ref7] Delaglio F, Grzesiek S, Vuister GW, Zhu G, Pfeifer J, Bax A. NMRPipe: a multidimensional spectral processing system based on UNIX pipes. J Biomol NMR. 1995:6(3):277–293. 10.1007/BF00197809. 8520220

[ref8] Gatti G, Casu B, Hamer GK, Perlin AS. Studies on the conformation of heparin by 1H and 13C NMR spectroscopy. Macromolecules. 1979:12(5):1001–1007. 10.1021/ma60071a044.

[ref9] Gildea RJ, Beilsten-Edmands J, Axford D, Horrell S, Aller P, Sandy J, Sanchez-Weatherby J, Owen CD, Lukacik P, Strain-Damerell C, et al. xia2.Multiplex: a multi-crystal data-analysis pipeline. Acta Crystallogr D Struct Biol. 2022:78(6):752–769. 10.1107/S2059798322004399. PMC9159281 35647922

[ref10] Han Y-H, Garron M-L, Kim H-Y, Kim W-S, Zhang Z, Ryu K-S, Shaya D, Xiao Z, Cheong C, Kim YS, et al. Structural snapshots of heparin depolymerization by heparin Lyase I^*^. J Biol Chem. 2009:284(49):34019–34027. 10.1074/jbc.M109.025338. PMC2797172 19801541

[ref11] Helmus JJ, Jaroniec CP. Nmrglue: an open source python package for the analysis of multidimensional NMR data. J Biomol NMR. 2013:55(4):355–367. 10.1007/s10858-013-9718-x. PMC3636164 23456039

[ref12] Hemker HC . A century of heparin: past, present and future. J Thromb Haemost. 2016:14(12):2329–2338. 10.1111/jth.13555. 27862941

[ref13] Hogwood J, Mulloy B, Lever R, Gray E, Page CP. Pharmacology of heparin and related drugs: an update. Pharmacol Rev. 2023:75(2):328–379. 10.1124/pharmrev.122.000684. 36792365

[ref14] Karlsson R, Chopra P, Joshi A, Yang Z, Vakhrushev SY, Clausen TM, Painter CD, Szekeres GP, Chen Y-H, Sandoval DR, et al. Dissecting structure-function of 3-O-sulfated heparin and engineered heparan sulfates. Sci Adv. 2021:7(52):eabl6026. 10.1126/sciadv.abl6026. PMC8694587 34936441

[ref15] Lebedev AA, Young P, Isupov MN, Moroz OV, Vagin AA, Murshudov GN. JLigand: a graphical tool for the CCP4 template-restraint library. Acta Crystallogr D Biol Crystallogr. 2012:68(4):431–440. 10.1107/S090744491200251X. PMC3322602 22505263

[ref16] Limtiaco JFK, Beni S, Jones CJ, Langeslay DJ, Larive CK. NMR methods to monitor the enzymatic depolymerization of heparin. Anal Bioanal Chem. 2011:399(2):593–603. 10.1007/s00216-010-4132-7. PMC3015211 20814667

[ref17] Liu CY, Su WB, Guo LB, Zhang YW. Cloning, expression, and characterization of a novel heparinase I from Bacteroides eggerthii. Prep Biochem Biotechnol. 2020:50(5):477–485. 10.1080/10826068.2019.1709977. 31900079

[ref18] Meneghetti MC, Hughes AJ, Rudd TR, Nader HB, Powell AK, Yates EA, Lima MA. Heparan sulfate and heparin interactions with proteins. J R Soc Interface. 2015:12(110):20150589. 10.1098/rsif.2015.0589. PMC4614469 26289657

[ref19] Mochizuki H, Otsuka Y, Kimata K. Establishment of a method for measuring the 3-O-sulfated structure in heparan sulfate. Glycobiology. 2025:35(12):cwaf068. 10.1093/glycob/cwaf068. 41123490

[ref20] Murshudov GN, Skubak P, Lebedev AA, Pannu NS, Steiner RA, Nicholls RA, Winn MD, Long F, Vagin AA. REFMAC5 for the refinement of macromolecular crystal structures. Acta Crystallogr D Biol Crystallogr. 2011:67(4):355–367. 10.1107/S0907444911001314. PMC3069751 21460454

[ref21] Poyer S, Denys A, El Omrani N, Ilse M-B, Allain F, Damme M, Lübke T, Daniel R. Unlocking rare 3-O-Sulfation patterns in Heparan Sulfate via ARSG-directed Regioselective Desulfation and HILIC-MS. Anal Chem. 2026:98(8):5938–5946. 10.1021/acs.analchem.5c05985. 41699463

[ref22] Rabenstein DL . Heparin and heparan sulfate: structure and function. Nat Prod Rep. 2002:19(3):312–331. 10.1039/b100916h. 12137280

[ref23] Santini F, Bisio A, Guerrini M, Yates EA. Modifications under basic conditions of the minor sequences of heparin containing 2,3 or 2,3,6 sulfated-glucosamine residues. Carbohydr Res. 1997:302(1–2):103–108. 10.1016/S0008-6215(97)00111-0.

[ref24] Sarrazin S, Lamanna WC, Esko JD. Heparan sulfate proteoglycans. Cold Spring Harb Perspect Biol. 2011:3(7):1–33. 10.1101/cshperspect.a004952. PMC3119907 21690215

[ref25] Shriver Z, Sundaram M, Venkataraman G, Fareed J, Linhardt R, Biemann K, Sasisekharan R. Cleavage of the antithrombin III binding site in heparin by heparinases and its implication in the generation of low molecular weight heparin. Proc Natl Acad Sci U S A. 2000:97(19):10365–10370. 10.1073/pnas.97.19.10365. PMC27030 10984532

[ref26] Tickle IJ, Flensburg C, Keller P, Paciorek W, Sharff A, Vonrhein C, Bricogne G . STARANISO (http://staraniso.globalphasing.org/cgi-bin/staraniso.cgi). Cambridge, United Kingdom: Global Phasing Ltd., 2016.

[ref27] Tovar AMF, Santos GRC, Capillé NV, Piquet AA, Glauser BF, Pereira MS, Vilanova E, Mourão PAS. Structural and haemostatic features of pharmaceutical heparins from different animal sources: challenges to define thresholds separating distinct drugs. Sci Rep. 2016:6(1):35619. 10.1038/srep35619. PMC5067489 27752111

[ref28] Vagin A, Teplyakov A. Molecular replacement with MOLREP. Acta Crystallogr D Biol Crystallogr. 2010:66(1):22–25. 10.1107/S0907444909042589. 20057045

[ref29] Wang Z, Arnold K, Dhurandahare VM, Xu Y, Pagadala V, Labra E, Jeske W, Fareed J, Gearing M, Liu J. Analysis of 3-O-Sulfated Heparan Sulfate using isotopically Labeled oligosaccharide Calibrants. Anal Chem. 2022:94(6):2950–2957. 10.1021/acs.analchem.1c04965. PMC9006171 35107975

[ref30] Wang Z, Patel VN, Song X, Xu Y, Kaminski AM, Doan VU, Su G, Liao Y, Mah D, Zhang F, et al. Increased 3-O-sulfated heparan sulfate in Alzheimer’s disease brain is associated with genetic risk gene HS3ST1. Sci Adv. 2023:9(21):eadf6232. 10.1126/sciadv.adf6232. PMC10219595 37235665

[ref31] Yamada S, Yoshida K, Sugiura M, Sugahara K, Khoo KH, Morris HR, Dell A. Structural studies on the bacterial lyase-resistant tetrasaccharides derived from the antithrombin III-binding site of porcine intestinal heparin. J Biol Chem. 1993:268(7):4780–4787. 10.1016/S0021-9258(18)53465-7. 8444855

