# Supplementary material for: Functional characterization of a 3-O-sulfate tolerant heparin lyase from Bacteroides eggerthii
Source: Glycobiology. 2026 Oct 13;36(11):cwag097. doi: 10.1093/glycob/cwag097 (PMC13714783; doi:10.1093/glycob/cwag097)
Supplement: Supplementary file 1 [file rev1_supp_behepi_cwag097(1).docx]

**Supporting Information for**

Functional characterization of a 3-O-sulfate tolerant heparin lyase from Bacteroides *eggerthii*

Taran K. Kandola PhD^1,2‡^; Courtney J. Mycroft-West PhD^3,4‡^; Marcelo A. Lima PhD^2^; Abigail Turner PhD^3,4^; Cristina Gardini BSc^5^; Elena Urso MSc^6^; Thomas A. Torode PhD^2^; Gavin J. Miller PhD^2,7^; Antonella Bisio PhD^6^; Edwin A. Yates PhD^2,6,8^; Marco Guerrini MSc^6^; Mark A. Skidmore PhD^1,2,8^*, Liang Wu PhD^3,9^*

1. Centre for Marine and Coastal Studies (CEMACS), Universiti Sains Malaysia, 11800 USM, Penang, Malaysia
2. Centre for Glycoscience Research and Training, Keele University, Newcastle-under-Lyme, Staffordshire, ST5 5BG, ST5 5BG, United Kingdom
3. The Rosalind Franklin Institute, Harwell Science and Innovation Campus, OX11 0QX, OX11 0QX, United Kingdom
4. Department of Biochemistry, University of Oxford, South Parks Road, Oxford, OX1 3QU, United Kingdom
5. Centro Alta Tecnologia Istituto di Ricerche Chimiche e Biochimiche ‘G. Ronzoni’ S.r.l., Via G. Colombo 81, 20133, Milan, Italy
6. Istituto di Ricerche Chimiche e Biochimiche ‘G. Ronzoni’, Via G. Colombo 81, 20133, Milan, Italy
7. Manchester Institute of Biotechnology, University of Manchester, 131 Princess Street, Manchester, M1 7DN, United Kingdom
8. Department of Biochemistry, Cell and Systems Biology, Institute of Structural, Molecular and Integrative Biology, University of Liverpool, Crown Street, Liverpool, L69 7ZB, United Kingdom
9. Department of Pharmacology, University of Oxford, Mansfield Road, Oxford, OX1 3QT, United Kingdom

^‡^ Taran K. Kandola and Courtney J. Mycroft-West contributed equally to this work.

*Corresponding authors

Liang Wu, The Rosalind Franklin Institute, Harwell Science and Innovation Campus, OX11 0QX, United Kingdom ✉ [liang.wu@rfi.ac.uk](mailto:liang.wu@rfi.ac.uk)

Mark A. Skidmore, Centre for Marine and Coastal Studies (CEMACS), Universiti Sains Malaysia, 11800 USM, Penang, Malaysia Email: ✉ [m.a.skidmore@liv.ac.uk](mailto:m.a.skidmore@liv.ac.uk)

## ***Table S1.*** *Mass spectrum assignment of the main peaks from Base Peak Chromatogram (BPC) of fondaparinux sodium digested with PhHepI. See also main text* ***Figure 3****.*

| Peak | m/z expt | z | m/z thr. | Error (ppm) | Assignment | Proposed structure | Proposed route of formation |
| --- | --- | --- | --- | --- | --- | --- | --- |
| 1 | 337.9848 | -1 | 337.9857 | 2.7 | A1,2,0 | A_NS6S_ | A🡪GA*IA |
| 2 | 514.0155 | -1 | 514.0178 | 4.5 | U2,2,0 | A_NS6S_-G | *Second step peeling from A_NS6S_-G-A_NS6S_(–H_2_O)* |
| 3 | 477.9947 | -1 | 477.9967 | 4.2 | ΔU2,2,0(–H_2_O) | ΔU-A_NS6S_(–H_2_O) | *First step peeling from ΔU-A_NS3S6S_* |
| 4 | 589.9768 | -1 | 589.9797 | 4.9 | ΔU2,3,0-OMe | ΔU_2S_-A_NS6S_-OMe | AGA*🡪IA |
| 5 | 407.9904 | -2 | 407.9912 | 2.0 | A3,4,0(–H_2_O) | A_NS6S_-G-A_NS6S_(–H_2_O) | *First step peeling from A_NS6S_-G-A_NS3S6S_* |
| 6 | 456.9741 | -2 | 456.9749 | 1.8 | A3,5,0(–H_2_O) | A_NS6S_-G-A_NS3S6S_ | AGA*🡪IA |

## ***Table S2.*** *Mass spectrum assignment of the main peaks from BPC of fondaparinux sodium digested with BeHepI. See also main text* ***Figure 3****.*

| Peak | m/z expt | z | m/z thr. | Error (ppm) | Assignment | Proposed structure | Proposed route of formation |
| --- | --- | --- | --- | --- | --- | --- | --- |
| 1 | 337.9859 | -1 | 337.9857 | 0.6 | A1,2,0 | A_NS6S_ | *A🡪GA*IA* |
| 2 | 514.0232 | -1 | 514.0178 | 10.5 | U2,2,0 | A_NS6S_-G | *Second step peeling from A_NS6S_-G-A_NS6S_(–H_2_O)* |
| 3 | 477.9971 | -1 | 477.9967 | 0.8 | ΔU2,2,0(–H_2_O) | ΔU-A_NS6S_(–H_2_O) | *First step peeling from ΔU-A_NS3S6S_* |
| 4a  4b | 705.1161  589.9803 | -1  -1 | 705.1158  589.9797 | 0.4  1.0 | ΔU2,3,0(+DBA)  ΔU2,3,0-OMe | ΔU-A_NS3S6S_  ΔU_2S_-A_NS6S_-OMe | *A🡪GA*🡪IA*  *AGA*🡪IA* |
| 5 | 407.9924 | -2 | 407.9912 | 2.9 | A3,4,0(–H_2_O) | A_NS6S_-G-A_NS6S_(–H_2_O) | *First step peeling from A_NS6S_-G-A_NS3S6S_* |
| 6 | 456.9759 | -2 | 456.9749 | 2.2 | A3,5,0(–H_2_O) | A_NS6S_-G-A_NS3S6S_ | *AGA*🡪IA* |

## ***Table S3.*** *Mass spectrum assignment of the main peaks from BPC of heparin decasaccharide digested with PhHepI. See also* ***Figure S6****.*

| Peak | m/z exp. | z | m/z thr. | Error (ppm) | Assignment | Proposed structure | Proposed route of formation |
| --- | --- | --- | --- | --- | --- | --- | --- |
| 1a  1b | 575.9632  705.1147 | -1  -1 | 575.9640  705.1158 | 1.4  1.6 | ΔU2,3,0  ΔU2,3,0(+DBA) | ΔU_2S_-A_NS6S_ | *ΔU_2S_-A_NS6S_-I-A_NAc6S_-G-A_NS3S6S_🡪I_2S_-A_NS6S_🡪I_2S_-A_NS6S_* |
| 2 | 605.0285 | -2 | 605.0286 | 0.2 | ΔU5,4,1 | ΔU_2S_-A_NS6S_-I-A_6S_-G | *Peeling from ΔU_2S_-A_NS6S_-I-A_NAc6S_-G-A_NS3S6S_* |
| 3 | 640.5402 | -2 | 640.5399 | 0.5 | ΔU4,6,0(+DBA) | ΔU_2S_-A_NS6S_-I_2S_-A_NS6S_ | *ΔU_2S_-A_NS6S_-I-A_NAc6S_-G-A_NS3S6S_🡪I_2S_-A_NS6S_-I_2S_-A_NS6S_* |
| 4 | 934.6488 | -2 | 934.6500 | 1.3 | ΔU6,7,1(+2DBA) | ΔU_2S_-A_NS6S_-I-A_NAc6S_-G-A_NS3S6S_ | *ΔU_2S_-A_NS6S_-I-A_NAc6S_-G-A_NS3S6S_🡪I_2S_-A_NS6S_-I_2S_-A_NS6S_* |

## ***Table S4.*** *Mass spectrum assignment of the main peaks from BPC of heparin decasaccharide digested with BeHepI. See also* ***Figure S6****.*

| Peak | m/z exp. | z | m/z thr. | Error (ppm) | Assignment | Proposed structure | Proposed route of formation |
| --- | --- | --- | --- | --- | --- | --- | --- |
| 1a | 496.0066 | -1 | 496.0074 | 1.6 | ΔU2,2,0 | ΔU-A_NS6S_ | *Peeling from ΔU-A_NS3S6S_* |
| 2a  2b | 575.9634  705.1147 | -1  -1 | 575.9640  705.1158 | 1.0  1.6 | ΔU2,3,0  ΔU2,3,0(+DBA) | ΔU-A_NS3S6S_  ΔU_2S_-A_NS6S_ | *ΔU_2S_-A_NS6S_🡪I-A_NAc6S_🡪G-A_NS3S6S_🡪I_2S_-A_NS6S_🡪I_2S_-A_NS6S_* |
| 3 | 468.0289 | -2 | 468.0288 | 0.2 | ΔU4,3,1(-1,6an) | ΔU_2S_-A_NS6S_-I-A_NAc_(-1,6an) | *Elimination from ΔU_2S_-A_NS6S_-I-A_NAc6S_* |
| 4 | 517.0128 | -2 | 517.0125 | 0.6 | ΔU4,4,1 | ΔU_2S_-A_NS6S_-I-A_NAc6S_  ΔU-A_NAc6S_-G-A_NS3S6S_ | *ΔU_2S_-A_NS6S_-I-A_NAc6S_🡪G-A_NS3S6S_-I_2S_-A_NS6S_-I_2S_-A_NS6S_*  *ΔU_2S_-A_NS6S_🡪I-A_NAc6S_-G-A_NS3S6S_🡪I_2S_-A_NS6S_-I_2S_-A_NS6S_* |

## ***Table S5.*** *Mass spectrum assignment of the main peaks from BPC of PMH digested with PhHepI. See also* ***Figure S8****.*

| Peak | m/z exp. | z | m/z thr. | Error (ppm) | Assignment | Proposed structure from reference data |
| --- | --- | --- | --- | --- | --- | --- |
| 1 | 496.0065 | -1 | 496.0062 | 0.6 | ΔU2,2,0 | ΔU-A_NS6S_ |
| 2 | 496.0065 | -1 | 496.0062 | 0.6 | ΔU2,2,0 | ΔU_2S_-A_NS_ |
| 3 | 575.9637 | -1 | 575.9630 | 1.2 | ΔU2,3,0 | ΔU_2S_-A_NS6S_ |
| 4 | 535.9849 | -2 | 535.9846 | 0.6 | ΔU4,5,0 | ΔU-A_NS6S_-G-A_NS3S6S_ |
| 5 | 535.9851 | -2 | 535.9846 | 0.9 | ΔU4,5,0 | ΔU_2S_-A_NS_-G-A_NS3S6S_ |
| 6 | 894.6707 | -2 | 894.6707 | 0.0 | ΔU6,6,1 | - |
| 7 | 999.2251 | -2 | 999.2247 | 0.4 | ΔU6,7,1 | - |

## ***Table S6.*** *Mass spectrum assignment of the main peaks from BPC of PMH digested with BeHepI. See also* ***Figure S8****.*

| Peak | m/z exp. | z | m/z thr. | Error (ppm) | Assignment | Proposed structure from reference data |
| --- | --- | --- | --- | --- | --- | --- |
| 1 | 496.0065 | -1 | 496.0062 | 0.6 | ΔU2,2,0 | ΔU-A_NS6S_ |
| 2 | 496.0065 | -1 | 496.0062 | 0.6 | ΔU2,2,0 | ΔU_2S_-A_NS_ |
| 3 | 575.9637 | -1 | 575.9630 | 1.2 | ΔU2,3,0 | ΔU_2S_-A_NS6S_ |
| 4 | 477.0337 | -2 | 477.0330 | 1.5 | ΔU4,3,1 | ΔU-A_NAc6S_-G-A_NS3S_ |
| 5 | 517.0118 | -2 | 517.0114 | 0.8 | ΔU4,4,1 | ΔU-A_NAc6S_-G-A_NS3S6S_ |
| 6 | 517.0118 | -2 | 517.0114 | 0.8 | ΔU4,4,1 | ΔU_2S_-A_NAc6S_-G-A_NS3S_ |
| 7 | 535.9849 | -2 | 535.9846 | 0.6 | ΔU4,5,0 | ΔU-A_NS6S_-G-A_NS3S6S_ |

## ***Table S7****. X-ray data collection and refinement statistics*

|  | Structure of heparinase I from *Bacteroides Eggerthii* in complex with calcium cofactor and delta-UA(1-4)Glc 3, 6, N-Sulfated disaccharide (PDB: 9GV9) | Structure of heparinase I from *Bacteroides Eggerthii* in complex with calcium cofactor (PDB: 9GRW) |
| --- | --- | --- |
| **Data collection** |  |  |
| Space group | C 1 2 1 | C 1 2 1 |
| Cell dimensions |  |  |
| a, b, c (Å) | 139.59, 91.56, 73.44 | 140.19, 91.49, 73.45 |
| α, β, γ (°) | 90, 95.59, 90 | 90, 95.46, 90 |
| Resolution (Å) | 76.45–1.72 (1.90–1.72) | 54.29–1.85 (1.88–1.85) |
| *R*_merge_ | 0.165 (1.045) | 0.387 (2.965) |
| *I* / σ*I* | 7.7 (1.7) | 6.4 (0.8) |
| Completeness (%) | 90.4 (63.4); (ellipsoidal cut-off) | 99.9 (98.5) |
| Redundancy | 6.7 (6.0) | 7.0 (7.1) |
|  |  |  |
| **Refinement** |  |  |
| Resolution (Å) | 76.45–1.72 | 54.29–1.85 |
| No. reflections | 56418 / 2859 | 78793 / 3925 |
| *R*_work_ / *R*_free_ | 0.20 / 0.24 | 0.21 / 0.25 |
| No. atoms |  |  |
| Protein | 5898 | 5921 |
| Ligand/ion | 171 | 177 |
| Water | 578 | 671 |
| *B*-factors |  |  |
| Protein | 19.52 | 16.66 |
| Ligand/ion | 28.34 | 45.52 |
| Water | 22.58 | 28.26 |
| R.m.s. deviations |  |  |
| Bond lengths (Å) | 0.0037 | 0.0048 |
| Bond angles (°) | 1.202 | 1.254 |

Values in parentheses are for highest-resolution shell.

## ***Figure S1.*** *BeHepI purified from E. coli by IMAC, SEC and IEX.*

## ***Figure S2.*** *COSY NMR spectrum of fondaparinux following digestion with BeHepI (ΔUA2S and ΔUA).*

## ***Figure S3.*** *TOCSY NMR spectrum of fondaparinux following digestion with BeHepI (ΔUA2S and ΔUA).*

## ***Figure S4.*** *TOCSY NMR spectrum of fondaparinux following digestion with BeHepI. Reducing end glucosamines are labelled within the figure.*

## ***Figure S5.*** *1-D ^1^H NMR spectra of fondaparinux sodium digestion by BeHepI over time, progressing from undigested substrate to fully digested, end-product (top).*

## ***Figure S6. a****) LC-MS BPC for decasaccharide digested by PhHep, showing principal sites of cleavage. A summary of main peak masses is given in* ***Table S3****.* ***b****) LC-MS BPC for decasaccharide digested by BeHep, showing principal sites of cleavage. A summary of main peak masses is given in* ***Table S4****.*

## ***Figure S7.*** ***a)*** *^1^H-^13^C HSQC spectrum of PMH following digestion with BeHepI at 5–6/93–109 ppm.* ***b)*** *^1^H-^13^C HSQC spectrum of PMH following digestion with BeHepI at 3.2–4.6/56–86 ppm.* ***c)*** *TOCSY NMR spectrum of PMH following digestion with BeHepI, showing coupling across Ared-A2*-A3*.*

## ***Figure S8. a****) LC-MS BPC for PMH digested by PhHep. A summary of main peak masses is given in* ***Table S5****.* ***b****) LC-MS BPC for PMH digested by BeHep. A summary of main peak masses is given in* ***Table S6****.*

## ***Figure S9.*** *Overlay of apo (green) and ligand bound BeHepI (blue) tertiary structures.*

## ***Figure S10.*** *BeHepI calcium binding site.*

## ***Figure S11.*** *REFMAC σ_A_-weighted mFo-DFc map levels for BeHepI ligand complex contoured to* ***a)*** *1.5σ (0.13 e^-^/Å^3^)* ***b)*** *2σ (0.25 e^-^/Å^3^), and* ***c)*** *3σ (0.38 e^-^/Å^3^).*

## ***Figure S12 a)*** *Multiple sequence alignment PhHepI. BeHepI and of BtHepI (PDB 9GV9) performed using Clustal Omega (Sievers et al., 2014) and visualized using Jalview (Waterhouse et al., 2009).* ***(b-d)*** *Comparison of Coulomb electrostatic potential maps of PhHepI (left - AF-Q05819-F1-v4), BtHep1 (middle - PDB 3IKW) and BeHepI (right - PDB 9GV9) respectively, calculated using the APBS plugin for PyMoL.*

# Supplemental References

Sievers F and Higgins DG. 2014. Clustal omega. Curr Protoc Bioinformatics. 48:3 13 11-13 13 16.

Waterhouse AM, Procter JB, Martin DM, Clamp M and Barton GJ. 2009. Jalview Version 2-a multiple sequence alignment editor and analysis workbench. Bioinformatics. 25:1189-1191.
